# 3-bromopyruvate ameliorate autoimmune arthritis by modulating Th17/Treg cell differentiation and suppressing dendritic cell activation

**DOI:** 10.1038/srep42412

**Published:** 2017-02-10

**Authors:** Takaichi Okano, Jun Saegusa, Keisuke Nishimura, Soshi Takahashi, Sho Sendo, Yo Ueda, Akio Morinobu

**Affiliations:** 1 Rheumatology and Clinical Immunology, Kobe University Graduate School of Medicine, Kobe, Japan; 2 Department of Clinical Laboratory, Kobe University Hospital, Kobe, Japan

## Abstract

Recent studies have shown that cellular metabolism plays an important role in regulating immune cell functions. In immune cell differentiation, both interleukin-17-producing T (Th17) cells and dendritic cells (DCs) exhibit increased glycolysis through the upregulation of glycolytic enzymes, such as hexokinase-2 (HK2). Blocking glycolysis with 2-deoxyglucose was recently shown to inhibit Th17 cell differentiation while promoting regulatory T (Treg) cell generation. However, 2-DG inhibits all isoforms of HK. Thus, it is unclear which isoform has a critical role in Th17 cell differentiation and in rheumatoid arthritis (RA) pathogenesis. Here we demonstrated that 3-bromopyruvate (BrPA), a specific HK2 inhibitor, significantly decreased the arthritis scores and the histological scores in SKG mice, with a significant increase in Treg cells, decrease in Th17 cells, and decrease in activated DCs in the spleen. *In vitro*, BrPA facilitated the differentiation of Treg cells, suppressed Th17 cells, and inhibited the activation of DCs. These results suggested that BrPA may be a therapeutic target of murine arthritis. Although the role of IL-17 is not clarified in the treatment of RA, targeting cell metabolism to alter the immune cell functions might lead to a new therapeutic strategy for RA.

Rheumatoid arthritis (RA) is characterized by synovial inflammation and hyperplasia, autoantibody production, and cartilage and bone destruction. Dendritic cells (DCs), monocytes, T cells, B cells, and neutrophils infiltrate the synovium and interact with each other and synovial fibroblasts to induce chronic synovitis^1^,^2^.

Pro-inflammatory cytokines are involved in the pathogenesis of RA^3^. In particular, interleukin (IL)-17 induces stromal cells to produce IL-6 and tumor necrosis factor (TNF)–α, macrophage activation, and osteoclast differentiation^1^. Therefore, IL-17 aggravates synovial inflammation and promotes cartilage and bone destruction^4^,^5^. In contrast, Foxp3 + regulatory T (Treg) cells promote immune tolerance and inhibit autoimmunity^6^. Thus, the IL-17-producing helper T (Th17)/Treg balance has an important role in RA development^7^.

Immune cells utilize different metabolic pathways in differentiation to and maintenance of their phenotypes^8^,^9^. Th17 cells rely mainly on glycolysis^10^, whereas Treg cells depend on fatty acid oxidation (FAO) and oxidative phosphorylation (OXPHOS)^9^. In addition, a recent report suggests that DC activation also requires an increase in glycolysis^11^, while FAO is important in tolerogenic DCs (tol-DCs)^12^. It is of note that both Th17 cells and activated DCs, which induce immune response, are dependent on glycolysis like cancer cells.

The first step in glycolysis is the phosphorylation of glucose, catalyzed by hexokinase (HK). There are four isoforms of HKs. While HK1 is ubiquitously expressed, HK2 is expressed at high levels in only a limited number of adult tissues, including adipose, skeletal, lung, and cardiac muscle tissue^13^. HK2 is considered the inducible HK form, because HK2 can be up-regulated approximately 100-fold in Th17 cells^10^. Therefore, HK2 is a key rate-limiting factor in the development of Th17 cells.

An inhibitor of all forms of HK, 2-deoxy-D-glucose (2-DG), is reported to ameliorate inflammation in experimental autoimmune encephalomyelitis (EAE) model mice by facilitating Treg-cell and suppressing Th17 cell differentiation^10^. In addition, 2-DG suppresses the DC activation induced by Toll like receptor 4 (TLR4) signaling^14^. Although the specific inhibition of HK2 is a more suitable therapeutic strategy, the effect of 3-bromopyruvate (BrPA), a specific HK2 inhibitor, on inflammatory disease has been little investigated. The effect of BrPA on Th17 cells or DCs is still unknown.

Here we investigated the effect of BrPA on the SKG mouse model, a well-established genetic model that exhibits many features of RA, including chronic destructive arthritis with a Th17 phenotype^15^. We show for the first time that BrPA ameliorates SKG arthritis, facilitates the differentiation of Treg cells, and suppresses Th17 cells. We also show that BrPA decreases the frequency of activated DCs. These results suggest that BrPA has dual effects on immune cells: it decreases proinflammatory immune cells, i.e., the Th17 cells and activated DCs, and increases anti-inflammatory immune cells, the Treg cells.

## Results

### Lymphocytes in the synovium of RA patients express HK2

**Figure 1:**
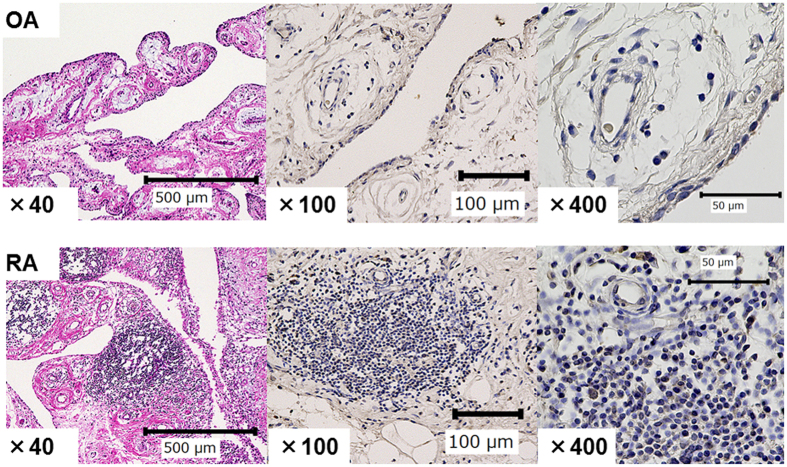
Lymphocytes express hexokinase-2 in the synovium of patients with rheumatoid arthritis. Synovial tissue was obtained from rheumatoid arthritis (RA) or osteoarthritis (OA) patients. OA (upper) and RA (lower) synovial tissue is shown. Synovium sections were stained with Hematoxylin and eosin (left; original magnification ×40). Synovium sections were stained with an anti-hexokinase-2 antibody (brown) and a nuclear stain (blue) (middle and right; original magnification ×100, ×400).

HK2 expression in the synovitis tissue of RA patients was examined by immunohistochemistry. We found that lymphocytes infiltrated the synovial tissue in RA patients and expressed HK2. In contrast, few lymphocytes infiltrated the synovium in OA patients, and they did not express HK2. The FLS in both the RA and OA synovial tissues expressed HK2 at a comparable level (Fig. 1).

### BrPA prevents arthritis progression in SKG mice

**Figure 2:**
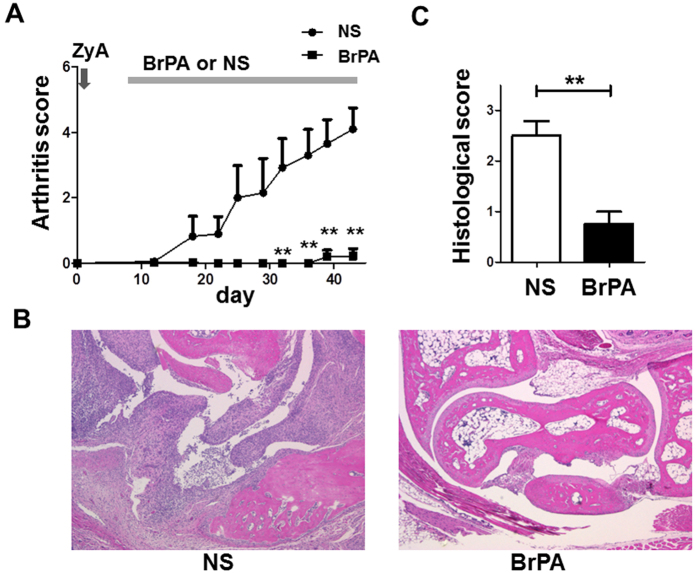
BrPA treatment prevents the progression of SKG mouse arthritis. Either 3-bromopyruvate (BrPA) (5 mg/kg) or normal saline (NS) was intraperitoneally administered to Zymosan A (ZyA)-treated arthritic SKG mice. Each group was 4 mice. (**A**) Clinical arthritis scores were determined for 43 days after ZyA injection. (**B**) The right hind paws of BrPA- or NS-treated SKG mice were evaluated for histopathologic change on day 43. Hematoxylin and eosin staining; original magnification ×40. (**C**) Histological arthritis scores were determined using the right hind paws of SKG mice in each group. Results in A and C are the mean ± SEM. *P < 0.05; **P < 0.01. n.s. = not significant (see Fig. 1 for other definitions).

To study the effect of BrPA on inflammatory arthritis, we first administered BrPA (5 mg/kg) via intraperitoneal injection once daily in arthritis SKG mice. BrPA treatment significantly decreased the severity of the arthritis in the SKG mice compared to the NS-treated control group (Fig. 2A). We next examined these mice histologically. BrPA significantly reduced the severity of synovial hyperplasia and pannus formation, and the extent of bone destruction (Fig. 2B, C). These results suggest that BrPA ameliorates the arthritis in SKG mice.

### BrPA reduces inflammation by increasing regulatory T cells and decreasing DCs

**Figure 3:**
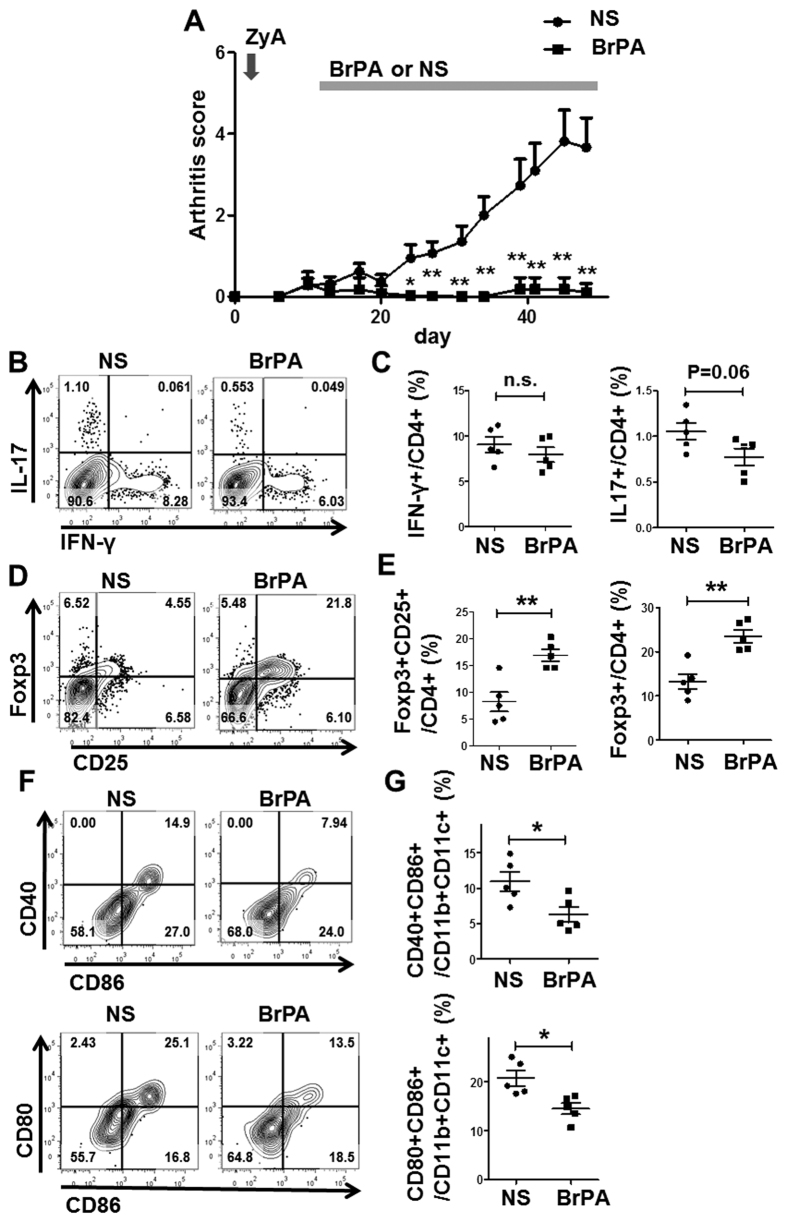
BrPA reduces inflammation by increasing Treg cells and decreasing DCs. Either BrPA (5 mg/kg) or NS was subcutaneously administered to ZyA-treated arthritic SKG mice. Each group was 5 mice. (**A**) Clinical arthritis scores were determined up to 48 days after ZyA injection. (**B, D**) Splenocytes from BrPA- or NS-treated arthritic SKG mice were stained with anti-CD4, anti-interferon (IFN)-γ, anti-interleukin (IL)-17, anti-Foxp3, and anti-CD25 antibodies, and analyzed by flow cytometry. (**C, E**) Frequency of IFNγ + , IL-17 + , Foxp3 + CD25 + , and Foxp3 + cells among the CD4 + cells in the spleen of BrPA- or NS-treated SKG mice, analyzed by flow cytometry. (**F, G)** Splenocytes from BrPA- or NS-treated SKG mice were stained with antibodies against CD11b, CD11c, CD80, CD86, and CD40 and analyzed by flow cytometry (left). Frequency of CD86 + CD40 + and CD86 + CD80 + DCs among the CD11b + CD11c + cells, analyzed by flow cytometry (right). Bars show mean ± SEM. *P < 0.05; **P < 0.01. n.s. = not significant (see Fig. 2 for other definitions).

Since the intraperitoneal injection of BrPA might have caused mild peritonitis, we injected BrPA subcutaneously to further define its effect on immune cells in the SKG mice. Daily subcutaneous injections of BrPA (5 mg/kg) also almost completely suppressed the arthritis development in the SKG mice (Fig. 3A). We did not find a significant effect of BrPA on the frequency of Th1 cells, but the Th17 cells tended to decrease in the spleens from BrPA-treated SKG mice (P = 0.06) (Fig. 3B, C). Furthermore, the percentages of both CD25 + Foxp3 + and Foxp3 + Treg cells were significantly increased in the BrPA-treated compared to NS-treated mice (Fig. 3D, E). DCs activated by LPS were previously reported to increase their expression of maturation markers, such as CD40, CD80, and CD86, which are essential costimulatory factors for T-cell activation^11^. We found that BrPA reduced the proportion of CD40 + CD86 + CD11c + CD11b + DCs (activated DCs), and CD80 + CD86 + CD11c + CD11b + DCs (Fig. 3F). These results suggested that BrPA ameliorates the inflammation in SKG arthritis by facilitating the differentiation of Treg cells and suppressing the activation of DCs.

### BrPA facilitates Treg-cell differentiation and suppresses Th17 cells

**Figure 4:**
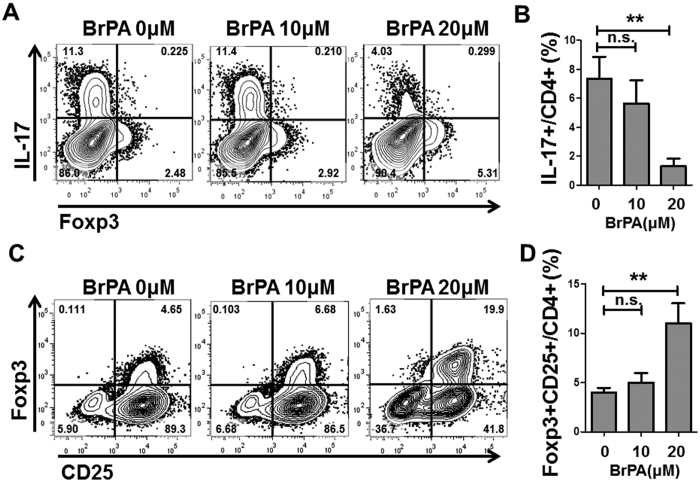
BrPA facilitates the differentiation of Treg cells and suppresses Th17 cells. CD4 + T cells were isolated from unimmunized SKG mice, and cultured for 5 days with pre-coated anti-CD3, anti-CD28, human IL-2, transforming growth factor (TGF)-β, anti-IFN-γ, and anti-IL-4, with and without BrPA for the last 3 days. (**A**,**C**) Cells were stained with anti-CD4, anti-IL-17, anti-Foxp3, and anti-CD25, and analyzed by flow cytometry. (**B**, **D**) The frequencies of IL-17 + and Foxp3 + CD25 + cells among CD4 + cells, analyzed by flow cytometry. Data are representative of 7 independent experiments with similar results. Bars represent mean ± SEM. *P < 0.05; **P < 0.01. n.s. = not significant (see Fig. 3 for other definitions).

We next investigated the effect of BrPA on Th17 cell differentiation. Inhibiting glycolysis with 2-DG was previously shown to decreases Th17 cell differentiation^10^. We cultured CD4 + T cells under Th17 conditions, and found that BrPA dose-dependently inhibited the differentiation of Th17 cells (Fig. 4A, B). We also demonstrated that BrPA facilitated the differentiation of Treg cells (Fig. 4C, D). These results confirmed that BrPA ameliorates the inflammation in SKG arthritis by facilitating the differentiation of Treg cells and suppressing that of Th17 cells.

### BrPA suppresses the differentiation of activated DCs

**Figure 5:**
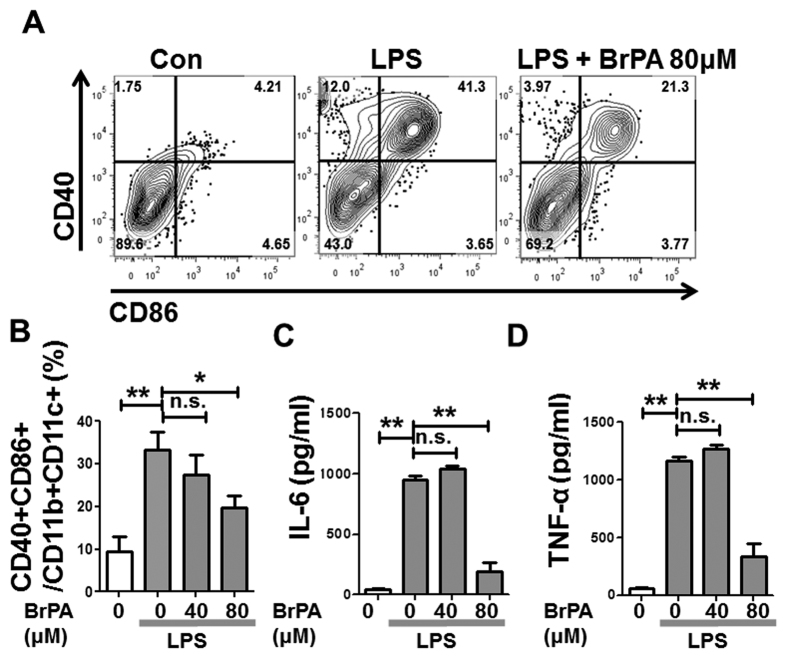
BrPA suppresses the differentiation of activated DCs. Bone marrow (BM) cells from unimmunized SKG mice were cultured with granulocyte-macrophage colony-stimulating factor (GM-CSF), IL-4, and lipopolysaccharide (LPS), and treated with and without BrPA. (**A**) Cells were stained with antibodies against CD11b, CD11c, CD86, and CD40, and analyzed by flow cytometry. (**B**) Frequency of CD40 + CD86 + cells among CD11b + CD11c + cells in each group, analyzed by flow cytometry. (**C**, **D**) Levels of IL-6, and tumor necrosis factor (TNF)-α in the culture supernatants from **B** measured by ELISA. All data are representative of 3 or more independent experiments with similar results. Bars show mean ± SEM. *P < 0.05; **P < 0.01. n.s. = not significant (see Fig. 4 for other definitions).

To further explore the effect of BrPA on DC activation, we performed *in vitro* experiments. While BrPA did not induce DC apoptosis or inhibit DC proliferation (Supplemental Figure S1), BrPA decreased the activated DCs in a dose-dependent manner (Fig. 5A, B). BrPA at 80 μM inhibited the LPS-induced maturation of BM-derived activated DCs. Activated DCs produce mainly IL-6 and TNF-α in the joints of RA patients^16^. ELISAs revealed decreased levels of these cytokines in the supernatant of 80 μM BrPA-treated cells (Fig. 5C, D). These result strongly suggested that BrPA ameliorates inflammation in SKG arthritis not only by facilitating the differentiation of Treg cells but also by suppressing the activation of DCs.

### BrPA halts the progression of ongoing arthritis

**Figure 6:**
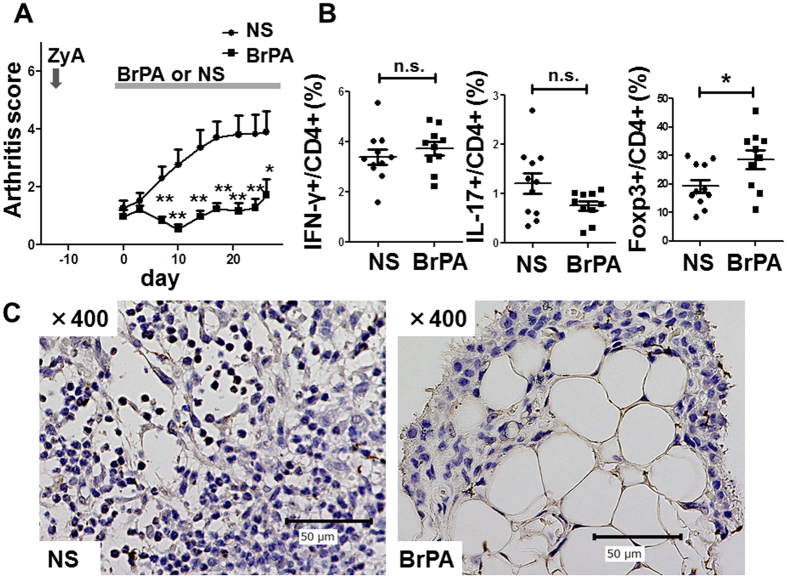
BrPA halts the progression of ongoing arthritis in SKG mice. ZyA-treated arthritic SKG mice were randomly divided into two groups on day 0 (mean arthritis score >1), and treated with BrPA or NS. Each group was 10 mice. (**A**) Clinical arthritis scores were determined for up to 4 weeks. (**B**) Frequencies of IFN-γ + , IL-17 + , and Foxp3 + cells among CD4 + cells in the spleen of BrPA-treated or NS-treated SKG mice, analyzed flow cytometry. Bars represent mean ± SEM. (**C**) Joint sections from BrPA-treated and NS-treated SKG mice were stained with an anti-hexokinase-2 antibody (brown) and nuclear stain (blue); original magnification ×400. *P < 0.05; **P < 0.01. n.s. = not significant (see Fig. 5 for other definitions).

To further explore the therapeutic potential of BrPA for arthritis, we studied its effect on ongoing arthritis in SKG mice. We found that arthritis did not progress in BrPA-treated mice even when BrPA was started after the onset of arthritis (Fig. 6A). BrPA also increased the frequency of Foxp3 + Treg cells in the spleen of these mice (Fig. 6B). Furthermore, we confirmed that the lymphocytes infiltrating the synovium of NS-treated mice expressed HK2 (Fig. 6C).

## Discussion

Here we showed the effect of BrPA on immune cells in inflammatory arthritis mice for the first time. BrPA ameliorated the autoimmune arthritis in SKG mice, facilitated the differentiation of Treg cells, and suppressed Th17 cells and suppressed the activation of DCs *in vivo* and *in vitro*.

Chronic inflammatory diseases, including RA, show altered metabolic profiles, such as increased peripheral insulin resistance (IR), which leads to the development of type 2 diabetes (T2DM) mellitus and cardiovascular disease (CVD)^17^,^18^. Also, chronic infection such as HIV and HCV show increase in IR, T2DM, dyslipidemia, and CVD risk^19^,^20^,^21^. Moreover, recent studies have revealed that inflammatory conditions are also associated with disturbance in cell metabolism such as higher requirement of glycolysis and OXPHOS for maturation of T cells and macrophages^8^,^9^. It has been reported that glucose transporter 1 expressions and glycolytic activity is increased in RA-FLS, and that glycolytic enzyme activity was elevated in CD4 + T cells and astrocytes in multiple sclerosis patients^22^,^23^,^24^.

Accordingly, improving metabolic disturbance may be a therapeutic strategy to reduce inflammation. A case control study revealed that acarbose, α- glucosidase inhibitor, prevented RA incidence in T2DM patient, and the drug prevented progression of arthritis in CIA mice^25^. A randomized open label control study showed that the combination of sitagliptin, a dipeptidyl peptidase-4 inhibitor, with ultraviolet phototherapy improved psoriasis more effectively than phototherapy alone^26^. Another observational study reported that sidagliptin decrease inflammation in HIV infection^27^. Also metformin ameriorates disease activity in CIA mice and IBD mice^28^,^29^. In a direct way, acute starvation reduced RA activity and T cell activation in the patients^30^. Interestingly, RA patients shared many up-regulated genes with T2DM patients, such as genes involved in classical complement pathway and activation of antigen-presenting cells, NK cells and Th17 cells^31^. These results supported the idea that there is a causal relationship between metabolic disturbance and chronic inflammation in a bidirectional way. Chronic inflammation affects cell metabolism, and metabolic profile in immune cells are critical for chronic inflammation. Our results clearly showed that metabolic pathway is one of the targets for the treatment of chronic inflammation.

IL-17 is the signature cytokine of the Th17 cell population, and is implicated in the pathogenesis of numerous autoimmune diseases including RA^1^. Glycolysis inhibition is a therapeutic strategy for RA. Bian *et al*. reported that blocking glycolysis with dichloroacetate (DCA), a pyruvate dehydrogenase kinase inhibitor, ameliorates autoimmune arthritis in the collagen-induced arthritis (CIA) model^32^. However, they did not study the effect of DCA on the Th17/Treg axis. Garcia-Carbonell *et al*. first reported that BrPA ameliorates arthritis in collagen-induced arthritic mice^22^. However, while they examined the effect of BrPA on RA fibroblast-like synoviocyts (FLS), they did not mention T cells or DCs. Shi *et al*. reported that inhibiting glycolysis with 2-DG ameliorates the disease in an EAE model by suppressing Th17 cell differentiation^10^. 2-DG inhibits all isoforms of HK, a family with 4 isoforms. Thus, it is unclear which isoform has a critical role in treatment of EAE model mice and in Th17 cell differentiation. Here we showed that the specific inhibition of HK2 was enough to facilitate the differentiation of Treg cells and inhibit Th17 cells. However further experiments are required to understand the mechanism behind the BrPA treatment with RA, because IL-17 blockade has not been proven effective in the treatment of rheumatoid arthritis.

We also showed for the first time that BrPA suppresses DC activation. TLR-stimulated BM-derived DCs have an increased glycolytic rate, increased lactate production, and decreased OXPHOS, and fatty acid oxidation (FAO)^33^. Everts *et al*. demonstrated that 2-DG inhibits glycolysis in monocytes and suppresses DC activation^14^. They found that signaling via the kinases TBK1, IKKε, and Akt is essential for TLR-induced increased glycolysis by promoting the association of HK2 with mitochondria^14^. We speculate that BrPA suppress DC activation in the similar way to that of 2-DG. Tolerogenic dendritic cells (tol-DCs) have important role in tolerance. Recently it has been reported that FAO is a key source of carbon during tol-DC differentiation^12^. Inhibition of glycolysis may lead to increase FAO in means of carbon source. Thus, BrPA may not only suppress DC activation, but also facilitate the differentiation of tolerogenic DCs. We did not identify tol-DC differentiation in our experiments because inhibition of glycolysis does not affect maturation markers expressed on tolerogenic DCs^34^. In order to prove this possibility, further experiments including functional assay of tol-DC is required.

DC and Th17 cells play a co-operative role in the arthritis. For example, GM-CSF produced by Th17 cells induces synovial inflammatory DCs, and IL-6 produced by DCs induces Th17 cell differentiation and an imbalance of the Th17/Treg axis^35^. Considering that both Th17 cells and DC activation are metabolically depend on glycolysis, BrPA is a rational therapeutic effect on inflammatory arthritis by metabolically suppressing the activationloop between Th17 and DC.

The mechanism for suppression of DC by BrPA activation is still unclear. In cancer cells, induced HK2 is mainly bound to the mitochondria and interacts with voltage-dependent anion channels (VDACs) located on the outer mitochondrial membrane. The HK2-VDAC interaction prevents tumor cells from undergoing apoptosis by inhibiting the release of cytochrome c from mitochondria through the VDAC^36^. BrPA can react with the sulfhydryl group of cysteine residues, to cause the HK2 dislocation from the mitochondria in cancer cells^37^. Thus BrPA is considered to cause tumor-cell death. However, we did not find any decrease in viable cells or any difference in cell proliferation between BrPA-treated and NS-treated DCs (Supplemental Fig. 1), while BrPA reduced the frequency of activated DCs in the spleen of SKG arthritis mice (Fig. 5A, B). These findings suggest that HK2 inhibition by BrPA suppresses DC activation via a different mechanism from induction of apoptosis. Millet *et al*. showed that GAPDH can inhibit translation of TNF-α by binding to its mRNA under low-glucose conditions^38^, but the mechanism for suppression of DC activation by BrPA needs investigation.

The mechanism by which glycolysis inhibition suppresses Th17 cells is well-investigated. Shi *et al*. demonstrated that Th17 cell differentiation requires IL-6 stimulation, which activates mTOR, leading to the overexpression of HIF-1α and glycolytic enzymes, and to an increase in glycolytic activity^10^, supporting the notion that up-regulation of T-cell glycolysis is not just a consequence of differentiation, but rather a necessary step to facilitate differentiation^39^. On the other hand, how glycolysis inhibition facilitates the differentiation of Treg cells remains unclear. The transcription factor Foxp3 interacts with ROR-γt and antagonizes its function^40^. Also, FoxO1 and FoxO3a co-operatively regulate the Foxp3 expression^41^,^42^. Thus, the fate of naïve T cells to differentiate into Th17 or Treg cells is considered to be regulated by the interaction between each transcription factors, ROR-γt, Foxp3, FoxO1, and FoxO3a. It has been shown that both 2-DG and BrPA inhibit glycolysis and increase the expression of FoxO3a in cancer cells^43^,^44^. Thus inhibition of glycolysis by BrPA may facilitate Treg cell differentiation by up-regulateing FoxO3a, leading to antagonising ROR-γt. Further investigations are required to determine the molecular events following inhibition of glycolysis.

## Conclusion

In this study, we demonstrated that BrPA ameliorates the autoimmune arthritis in SKG mice by facilitating the differentiation of Treg cells and by suppressing Th17 cells and activated DCs. Further investigation of the mechanisms of glycolysis inhibition in immune cells will reveal clues for new metabolic therapies for RA.

## Methods

### Ethical Provisions

The methods were carried out in accordance with the approved guidelines and regulations. After approval of the local ethical committees at Kobe University Hospital in Kobe (Japan), and written informed consent was obtained from all patients included in the study. All animal experiments were approved by the Institutional Animal Care and Use Committee of Kobe University.

### Animals and reagents

SKG mice were obtained from CLEA Japan, Inc. Mice were housed in the Kobe University animal facility at a constant temperature, with laboratory chow and water provided ad libitum. All procedures were carried out in accordance with the recommendations of the Institutional Animal Care Committee of Kobe University. BrPA, Zymosan A (ZyA), lipopolysaccharide (LPS), Phorbol 12-myristate 13-acetate (PMA), and Ionomycin (Iono) were from Sigma-Aldrich. RPMI 1640 (Wako Pure Chemical Industries) with 10% fetal bovine serum (FBS) (MP Biomedicals), 1% penicillin–streptomycin (P/St) (Lonza Walkersville), and 50 μM 2-mercaptoethanol (Sigma-Aldrich) were used for all cell cultures (culture medium). Normal saline (NS) was from Otsuka Pharmaceutical. All cytokines and antibodies added to cell cultures were from R&D Systems except for anti-CD3 (BD Biosciences), anti-CD28 (BD Biosciences), and recombinant murine granulocyte-macrophage colony-stimulating factor (GM-CSF) (Peprotec).

### Mouse arthritis model

Arthritis was induced in SKG mice by the intraperitoneal injection of 2 mg ZyA, as previously described^15^. Arthritis developed 2 to 3 weeks later. The development and severity of arthritis were assessed using a previously described system for scoring clinical arthritis^45^. We administered 5 mg/kg intraperitoneal BrPA (each group was 4 mice) in a prevention study. Then we administered 5 mg/kg subcutaneous BrPA (each group was 5 mice) for another prevention experiment. Finally, we examined the effect of 5 mg/kg BrPA on going arthritis in SKG mice (each group was 5mice).

### Histology

The hind paws of the mice were removed, fixed in 4% paraformaldehyde, decalcified in EDTA, embedded in paraffin, sectioned, and stained with hematoxylin and eosin (H.E.). Histologic evaluation was performed using a previously described scoring system, in which 0 = no inflammation, 1 = slight thickening of the synovial cell layer and/or some inflammatory cells in the sublining, 2 = thickening of the synovial lining, infiltration of the sublining, and localized cartilage erosions, and 3 = infiltration of the synovial space, pannus formation, cartilage destruction, and bone erosion^45^.

### Th17 cell differentiation

The CD4 + T cells were isolated from single-cell suspensions prepared from the splenocytes of unimmunized SKG mice, using a biotinylated mAb against CD4, streptavidin-coated magnetic beads, and a manual MACS system (all from Miltenyi Biotec), according to the manufacturer’s protocol. The CD4 + T cells were cultured for 5 days in culture medium at 37 °C under Th17 conditions: pre-coated 10 ng/ml anti-CD3, pre-coated 5 ng/ml anti-CD28, 10 ng/ml human IL-2, 10 ng/ml IL-6, 2.5 ng/ml TGF-β, 2.5 μg/ml anti-IFN-γ, and 2.5 μg/ml anti-IL-4. BrPA at 0, 10, or 20 μM was added for the last 3 days.

### DC differentiation

Bone marrow (BM) cells were obtained from unimmunized SKG mice and washed twice with PBS. The erythrocytes were then lysed by ACK Lysing Buffer (Lonza). The cells were filtered through a cell strainer and centrifuged at 1,500 rpm for 5 minutes at 4 °C. The BM cells were cultured at 1000 cells/μl, in culture medium at 37 °C, under DC conditions: 20 ng/ml GM-CSF, 10 ng/ml IL-4 (for 3 days), and 1 μg/ml LPS (for 1 day). The BM cells were also treated with BrPA (0, 40, 80 μM). The control group was cultured with GM-SCF and IL-4 only.

### ELISA

BM cells were cultured under DC conditions, with BrPA (0, 40, 80 μM), and then the IL-6 and TFN-α in the supernatant were analyzed by sandwich ELISA (R&D Systems), according to the manufacturer’s protocol, and by optical density measured at 450 mm with a microplate reader (Thermo).

### Flow cytometry

The following antibodies were used for flow cytometry: anti-CD4 (RM4-4), anti-CD11c (N418), anti-CD86 (GL1), anti-CD40 (1C10), anti- IFN-γ (XMG1.2), anti-IL-4 (BVD6-24G2), anti-IL-17 (eBIo17B7: all from eBioscience), anti-CD25 (3C7 from BD), anti-Foxp3 (150D from BioLegend), and anti-CD11b (M1/70 from BioLegend). For cytokine staining, sample cells were stimulated in culture medium containing10 ng/ml PMA and 1 ng/ml Iono (6 h), with 3 μg/ml brefildin A (eBioscience 00-4506) for the last 2 h, then treated with the Intracellular Fixation & Permeabilization Buffer Set (eBioscience 88-8824-00), according to the manufacturer’s protocol. For Foxp3 staining, samples were treated with the Foxp3 buffer set (BioLegend 421403). All samples were measured on a BD FACSVerse analyzer, and data were analyzed using Flowjo software.

### Immunohistochemistry

Synovial tissue samples were obtained from RA and osteoarthritis (OA) patients who had undergone joint replacement surgery. All of the RA patients fulfilled the American College of Rheumatology 1987 criteria. Tissue array blocks were cut into 4-μm-thick sections, which were deparaffinized in a dry oven at 60 °C for 1 h. To detect HK2 immunoreactivity, samples were immersed in Target Retrieval Solution (Dako) and incubated at 95 °C for 30 min for antigen retrieval. Endogenous peroxidase activity was blocked with 0.3% hydrogen peroxide for 30 min. After protein blocking for 1 h, the samples were incubated with a 1:50 dilution of goat polyclonal anti-HK2 antibody (C-14; Santa Cruz Biotechnology) overnight at 4 °C. The secondary antibody was then applied with the ImmunoCus ABC Staining System (Santa Cruz Biotechnology), according to the manufacturer’s protocol. The joints of SKG mice were stained using the same protocol described for the human samples.

### Statistical analysis

Statistical analyses and graphical presentations were done using GraphPad Prism version 5. Significance was calculated using an unpaired two tailed Student’s t-test. p < 0.05 was considered statistically significant. Values were expressed as the mean ± SEM. All data shown represent results from three or more independent observations.

## Additional Information

**How to cite this article**: Okano, T. *et al*. 3-bromopyruvate ameliorate autoimmune arthritis by modulating Th17/Treg cell differentiation and suppressing dendritic cell activation. *Sci. Rep.* **7**, 42412; doi: 10.1038/srep42412 (2017).

**Publisher's note:** Springer Nature remains neutral with regard to jurisdictional claims in published maps and institutional affiliations.

## Supplementary Material

Supplemental Figure S1
