# Supplementary material for: 3-bromopyruvate ameliorate autoimmune arthritis by modulating Th17/Treg cell differentiation and suppressing dendritic cell activation
Source: Sci Rep. 2017 Feb 10;7:42412. doi: 10.1038/srep42412 (PMC5301239; doi:10.1038/srep42412)
Supplement: Supplemental Figure S1 [file srep42412-s1.pdf]

3-bromopyruvate ameliorate autoimmune arthritis by modulating Th17/Treg cell differentiation and suppressing dendritic cell activation

Authors: Takaichi Okano[1], Jun Saegusa[2], Keisuke Nishimura[1], Soshi Takahashi[1], Sho Sendo[1], Yo Ueda[1], *Akio Morinobu[1].

1 Rheumatology and Clinical Immunology, Kobe University Graduate School of Medicine, Kobe, Japan

2 Department of Clinical Laboratory, Kobe University Hospital, Kobe, Japan

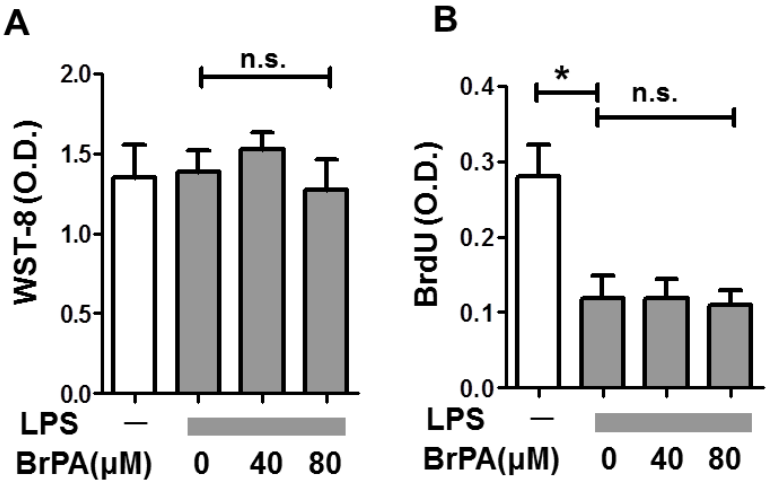

Supplemental figure S1. BrPA has no effect on cell survival and proliferation.

Bone marrow cells from unimmunized SKG mice were cultured with GM-CSF, IL-4, and LPS, and treated with and without 3-bromopyruvate **A,** Viable cell number was determined by WST-8 assay. **B,** Cell proliferation was analyzed by BrdU assay.
